# Spectroscopic methods for the simultaneous determination of amlodipine besylate and Hydrochlorothiazide in their binary mixture and pharmaceutical dosage form

**DOI:** 10.1038/s41598-025-33191-4

**Published:** 2026-02-02

**Authors:** Mohamed Badrawy, Omar M. El-Abassy, Israa M. Nour, Amr S. Eissa

**Affiliations:** Pharmaceutical Chemistry Department Faculty of Pharmacy, Egyptian Russian University, Badr City, 11829 Cairo Egypt

**Keywords:** AGREE, Amlodipine, Hydrochlorothiazide, Spider diagram, MOGAPI, Chemistry, Environmental sciences

## Abstract

The development of environmentally friendly analytical methods is important in the current scientific landscape. This study presents an investigation into several green spectrophotometric methods for the determination of amlodipine besylate and hydrochlorothiazide in both their pure binary mixtures and pharmaceutical formulations. A spectrophotometric method is proposed for the selective determination of amlodipine besylate in the presence of hydrochlorothiazide at 366 nm, where no interference from hydrochlorothiazide was observed. Additionally, a novel five different spectrophotometric approaches were applied for the determination of hydrochlorothiazide: the dual wavelength method, first derivative method, ratio difference method, and ratio derivative method, as well as Fourier Self-Deconvolution method. Amlodipine besylate’s linearity range was 5–30 µg/mL, whereas hydrochlorothiazide’s was 3–18 µg/mL reaching limits of detection of 1.238, 0.906, 0.825, 0.540, 0.779 and 0.805 µg/mL, respectively for all the proposed methods. The proposed methods were validated in accordance with ICH international guidelines, including full statistical analysis and comprehensive environmental evaluation using multiple assessment tools as The Analytical GREEnness Calculator, the Modified Green Analytic Procedure Index, spider diagram, and green analytical selection tool.

**Supplementary Information:**

The online version contains supplementary material available at 10.1038/s41598-025-33191-4.

## Introduction

**Fig. 1:**
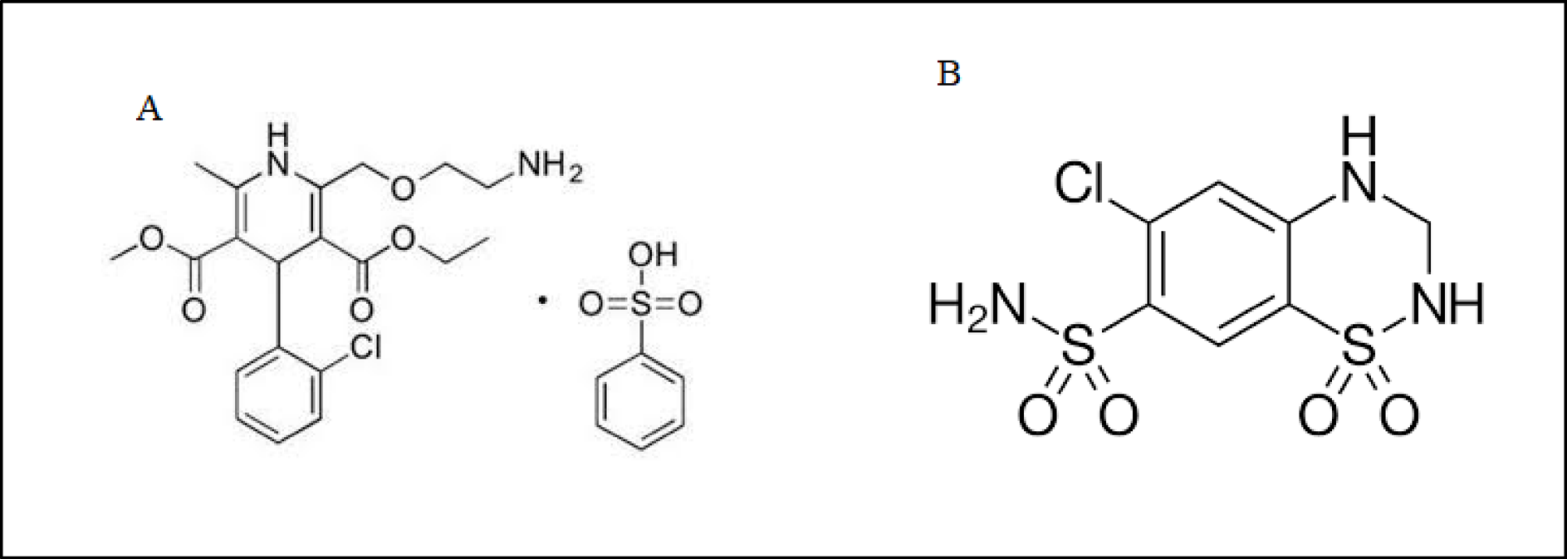
Chemical structure of (**A**) amlodipine besylate and (**B**) hydrochlorothiazide.

Hypertension, also referred to as high blood pressure, is a chronic medical condition characterized by the persistent elevation of blood pressure in the arteries. High blood pressure typically does not present noticeable symptoms. However, it is a significant risk factor for stroke, coronary artery disease, heart failure, atrial fibrillation, peripheral arterial disease, vision impairment, chronic kidney disease, and dementia. Hypertension is a leading cause of premature mortality worldwide^1–6^. Many drugs have been suggested for treatment of hypertensive either in single medication or combination of more than one drug. Amlodipine besylate (AMB) (3-Ethyl 5-methyl (4RS)−2-[(2-aminoethoxy)methyl]−4-(2chlorophenyl)−6-methyl-l,4dihydropyridine-3,5-dicarboxylate benzenesulfonate.) is a calcium channel blocker^7^. AMB is used as an antihypertensive for chronic stable angina, vasospastic angina, and Angiographically documented coronary artery disease alone or in combination with other drugs^8–11^. Hydrochlorothiazide (HYD) (6-Chloro-3,4-dihydro-2 H-l,2,4-benzothiadiazine-7 sulfonamide 1,I-dioxide.) is a thiazide diuretic^7^. HYD is used for the treatment of chronic hypertension, management of peripheral edema, and prevention of calcium nephrolithiasis^12–15^. A mixture of AMB and HYD Fig. 1 was approved from FDA for treatment of hypertension and its related condition^16,17^.

Although literature review shows many methods reported for determination of suggested drugs in mixtures with other drugs^18–28^. Only a few spectrophotometric techniques have been proposed for determining the levels of HYD and AMB in binary mixtures^20,29–31^. The spectroscopic techniques employed for the quantification of the target drugs with other drugs are either overly complex or inadequate for their simultaneous determination in a binary mixture. Chromatographic techniques, in contrast, are less eco-friendly since they are time-consuming, expensive, and need higher amounts of organic solvents like acetonitrile and methanol^19–23^.

Numerous medicinal substances have been quantified and analyzed using UV spectroscopy. This method offers distinct advantages over separation-based techniques, including its simplicity and short analysis time. Additionally, it typically requires a low volume of analytical solvents, making it a cost-effective approach from an economic perspective and more environmentally friendly. Even though spectrum overlaps are often encountered when using this method for combination examination, post-acquisition mathematics calculations may help to overcome these problems. Among them are univariate procedures, such obtaining ratio spectra using divisors, followed by additional processing of signals approaches such as derivatives, ratio differences, and mean centering approaches^32–35^.

The aim of this study is to develop environmentally friendly, cost-effective, and time-efficient spectroscopic analytical methods named (zero crossing method for determination of AMB and dual wavelength (DW) first derivative (D^1^), ration difference (RD), first ratio derivative (RD^1^) and Fourier self-deconvoluting (FSD) methods for the determination of (HYD), whether in their binary mixtures in pure form or within their pharmaceutical formulation. These methods are intended to be suitable for application in quality control laboratories and the pharmaceutical industry. The use of environmentally friendly analytical methods is of great importance for the preservation of the ecological system. This principle was carefully considered in the design of the present study, particularly through the use of water as the sole solvent, rendering the method highly eco-friendly. Unlike previous research that used methanol, which is more dangerous and expensive than water^36,37^. A number of evaluation techniques, including Analytical GREEnness Calculator (AGREE), Modified Green Analytic Procedure Index (MOGAPI), spider diagrams, and the green analytical selection tool, which demonstrated exceptional greenness, were used to further examine the environmental efficiency of the suggested approaches.

The proposed spectrophotometric methods offer several advantages over previously reported methods for the simultaneous determination of the two drugs in their binary mixture. They are characterized by simplicity, directness, and ease of application, as amlodipine can be determined using the zero-order spectrum method. Moreover, these methods are sustainable and environmentally friendly compared to other analytical approaches that rely on hazardous organic solvents. The environmental compatibility of the proposed procedures was evaluated, and since water was used as the diluting solvent, the most eco-friendly solvent available, the methods comply well with green analytical principles.

In addition, compared with previously reported methods developed for the determination of these drugs in ternary mixtures with other compounds, the proposed methods are superior in selectivity and practicality. Earlier methods were either unsuitable for determining the two drugs without the third due to spectral interference or involved complex mathematical manipulations, limiting their routine application.

## Experimental

###  Apparatus and software

All spectroscopic measures have been carried out employing a JASCO V-630 dual-beam spectrophotometer with ultraviolet (UV) light (JASCO, Japan), which contained 10 mm paired quartz cuvettes for measuring. Data acquisition was accomplished using the bundled software, Spectra Manager II, with the following parameters: a spectral bandwidth of 2 nm, a scanning speed up to 800 nm/min, and a data interval of 1 nm. A sonicator (DAIHAN WUC-A01H, USA) was employed. The weighing operations were performed using an analytical balance, model SA 210D (Scientech, USA). Microsoft Excel was utilized for data processing, including calibration, regression equation calculation, and statistical analysis (Student’s *t*-test and *F*-test). ANOVA and Tukey’s additional several tests of comparison were carried out using Minitab software.

### Chemical and reagent

Pure standard powders of AMB and HYD were kindly provided by Pfizer Pharmaceuticals, Cairo, Egypt, with certified purities of 99.75% and 99.30%, respectively. Amvasc plus 10/12.5 tablets (Batch No. NDC70347-0815-30), each claimed to contain 10 mg amlodipine and 12.5 mg of HYD, were manufactured by Unilab Pharmaceuticals Inc., Philippine. Double distilled water was used throughout the study.

### Standard solution

Individual stock standard solutions of AMB and HYD were prepared in water at a concentration of 100 µg/mL. Using the same solvent, these stock solutions were further diluted as required to obtain the desired concentrations.

### Procedures

#### Calibration curve

Various aliquots of the standard solutions for AMB and HYD were put into 10-mL volumetric flasks and filled to the highest point with water to obtain concentrations of 5 to 30 and 2 to 18 µg/mL for AMB and HYD, respectively. Using water as a blank, the absorption spectra (between 200 and 400 nm) of various solutions were captured and saved on the computer.

##### Zero crossing

The absorption of AMB at 366 nm was recorded and plotted against AMB concentration for construction of calibration curve used for determination of AMB.

##### Dual wavelength (DW)

HYD measurements were made by measuring the difference in absorption of the preserved spectra at 229 and 247 nm.

##### First derivative (D^1^)

The first derivative corresponding to HYD and AMB spectra (Δλ = 3 nm) was recorded. The amplitude value was measured at 235 nm for HYD (AMB is zero crossing) and plotted against HYD concentrations for construction of calibration curve used for determination of HYD.

##### Ratio difference (RD)

The HYD preserved spectra of absorption have been divided by implementing the AMB divisor (5 µg/mL). The difference in peak amplitude (ΔP) between 273 and 225 nm was recorded and plotted against HYD concentration for construction of calibration curve used for determination of HYD.

##### First ratio derivative (RD^1^)

The HYD preserved spectra of absorption have been divided by implementing the AMB divisor (5 µg/mL) and first derivative was recorded at 280 nm was recorded and plotted against HYD concentration for construction of calibration curve used for determination of HYD.

##### Fourier self-deconvoluting (FSD)

Each drug spectrum was deconvoluted by employing the full width at half maximum value (FWHM) of 60 and the Fourier deconvoluted function included in the spectrophotometer program. At 315 nm, when the absorption of AMB was zero, the amplitude of HYD was detected. In order to determine HYD, a graph of calibration was constructed that plotted the amplitudes of the deconvoluted spectra of HYD at 315 nm against the appropriate concentrations. The equation used for regression was then calculated.

#### Examination of mixtures made in a lab

Transferring aliquots of AMB and HYD from standard working solutions (100 µg/mL) into 10-mL volumetric flasks was done precisely. Following that, the quantities have been adapted with water to create lab mixes with varying doses of the two medications (10:5, 5:10, 10:10, 20:10, and 10:12.5 µg/mL) for AMB and HYD, respectively. After recording each laboratory-made combination’s zero-order spectrum versus water and storing it in the computer, the standard method was followed.

#### Utilizing the established approaches for AMB and HYB analysis

To extract the active ingredients from tablet excipients, ten tablets of Amvasc plus 10/12.5 (10 mg AMB/12.5 mg HYD) were ground and accurately weighed. After carefully weighing equivalent weight of one tablet and transferring it to a 100 mL volumetric flask, 50 mL of water was inserted and the mixture was ultrasonically treated for 30 min. The volume underwent filtering subsequent to cooling, and the generated solution was finished to create a stock solution that was marked as having 125 µg/mL of HYD and 100 µg/mL of AMB. By further diluting with water, various concentrations of AMB and HYD within the linearity limits were achieved. Using the relevant regression equations, the suggested techniques were used to perform a quantitative examination of the mentioned medications within the pharmaceutical formulation.

## Results and discussion

### Spectral characteristics

**Fig. 2:**
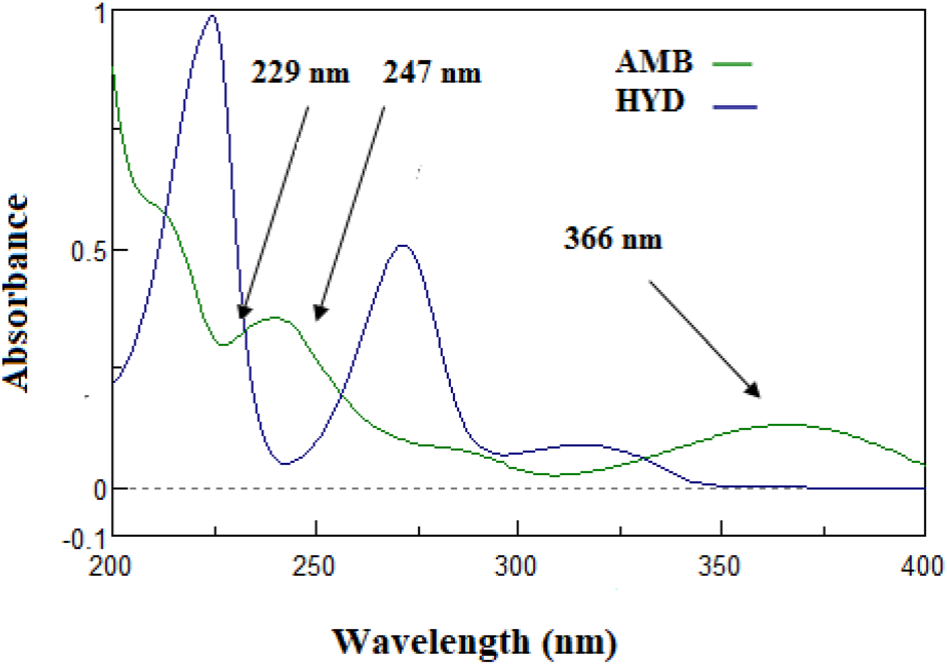
Zero-order absorption spectra of 10μg/mL of AMB, 10 μg/ml of HYD in water shows zero crossing of hydrochlorothiazide at 366 nm.

While AMB may be determined at 366 nm, where HYD is zero crossing, Fig. 2 shows severe spectral overlapping, which makes it more difficult to determine HYD directly from their zero-order absorption spectra. Therefore, the simultaneous quantification of HYD in binary mixture with AMB presents a considerable challenge. In order to solve this, five new and simple spectrophotometric techniques were presented, each using a distinct analytical strategy to accurate quantify the HYD in a combination containing AMB while determining AMB at 366 nm.

#### Zero crossing

**Fig. 3:**
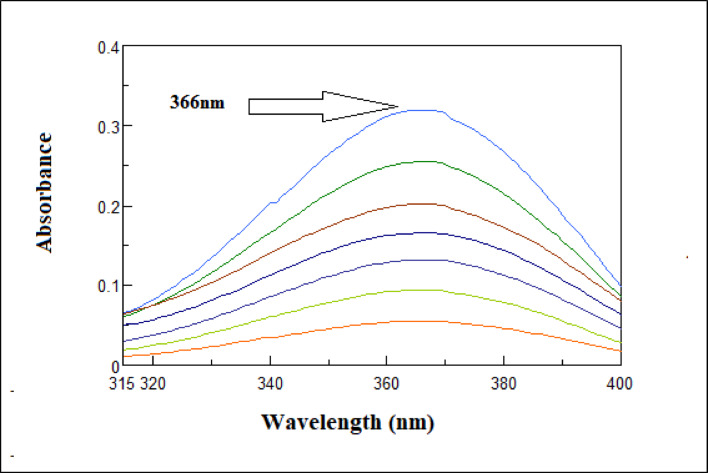
Zero order absorption spectrum of AMB (5–30 µg/mL).

AMB and HYD’s zero absorption spectra revealed severe overlap, making it impossible to determine them directly and simultaneously. AMB’s λ max at 366, however, indicates no interference with HYD (Fig. 2, 3).

#### Dual wavelength

A simple and effective technique for the selective identification of substances in their binary mixes utilizing zero-order absorption spectra is provided by the developed dual-wavelength spectrophotometric methodology^35^. To achieve the best possible selectivity and sensitivity, the right wavelength selection is essential. Various wavelength combinations were evaluated, and the most favorable results were obtained by measuring the absorbance differences at 229 nm and 247 nm for determination of HYD while the difference in absorption for AMB is zero (as shown in Fig. 2).

#### First derivative (D^1^)

**Fig. 4:**
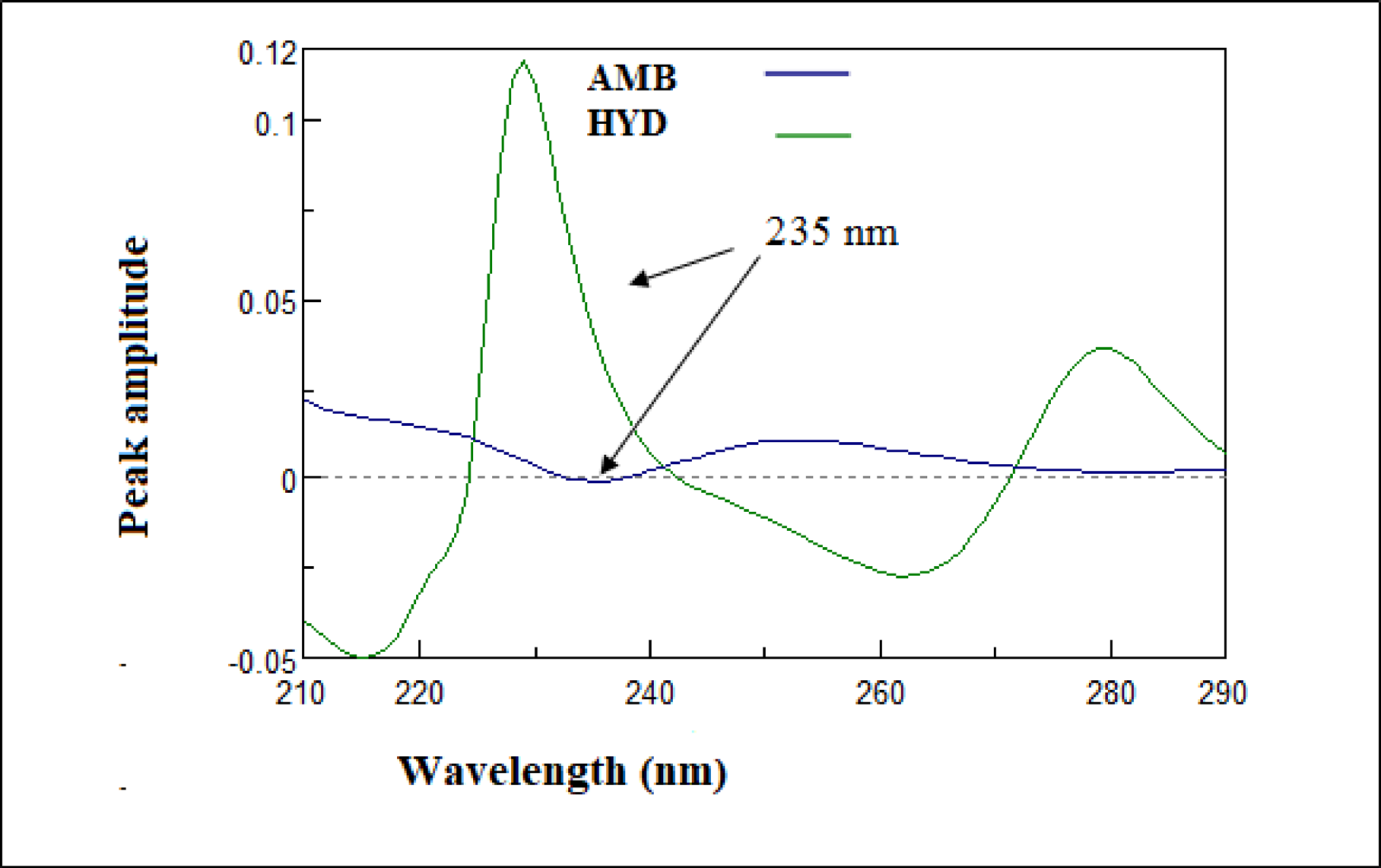
First derivative spectra of AMB (10 µg/mL) and HYD (10 µg/mL) using water as blank.

**Fig. 5:**
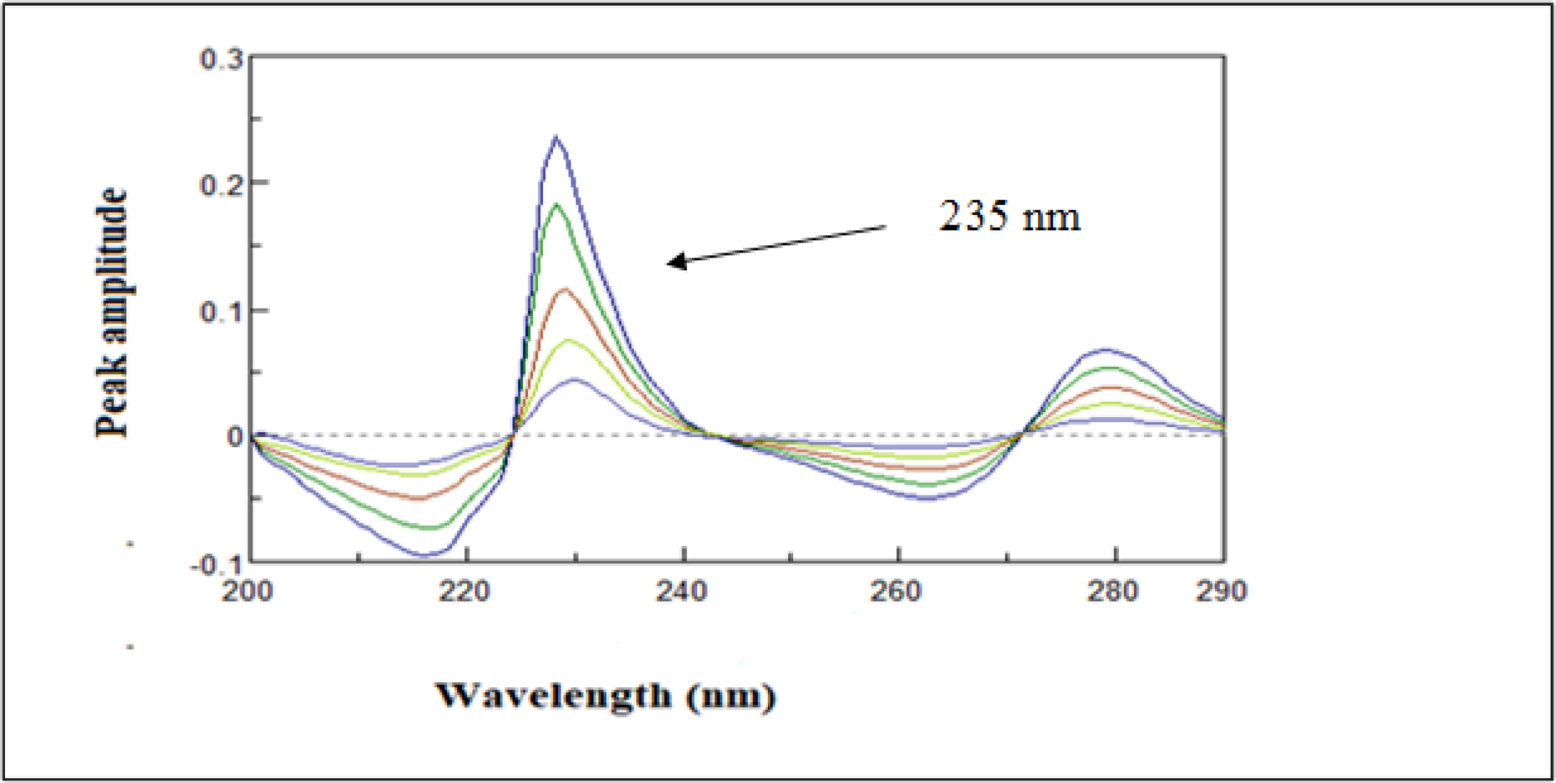
First derivative spectra of HYD (3–18 µg/mL) using water as blank.

The proposed derivative spectrophotometric method is based on calculating the first derivatives of the absorption spectra for HYD and AMB. Quantification is achieved by selecting wavelengths at which the analyte of interest exhibits a measurable peak while the interfering compound crosses the zero point, as shown in Fig. 4, thereby minimizing spectral interference, as illustrated in Fig. 5. Specifically, amplitude measurements were performed using a wavelength increment (Δλ) of 1 nm and a scale factor of 3. HYD was determined using the first-order derivative at 235 nm.

#### Ratio difference (RD)

Target medication concentration is precisely proportional to the difference among two identical spots on the resultant ratio spectra^38,39^. Each drug’s absorption spectrum is divided by the other medications’ spectra, which is utilized as a divisor to create these spectra. To guarantee accurate outcomes, choosing the right divisor is a crucial step that has to be carefully tuned. After testing a range of divisor concentrations, it was determined that 5 µg/mL was the most appropriate since it prevented noise from the other medication.

**Fig. 6:**
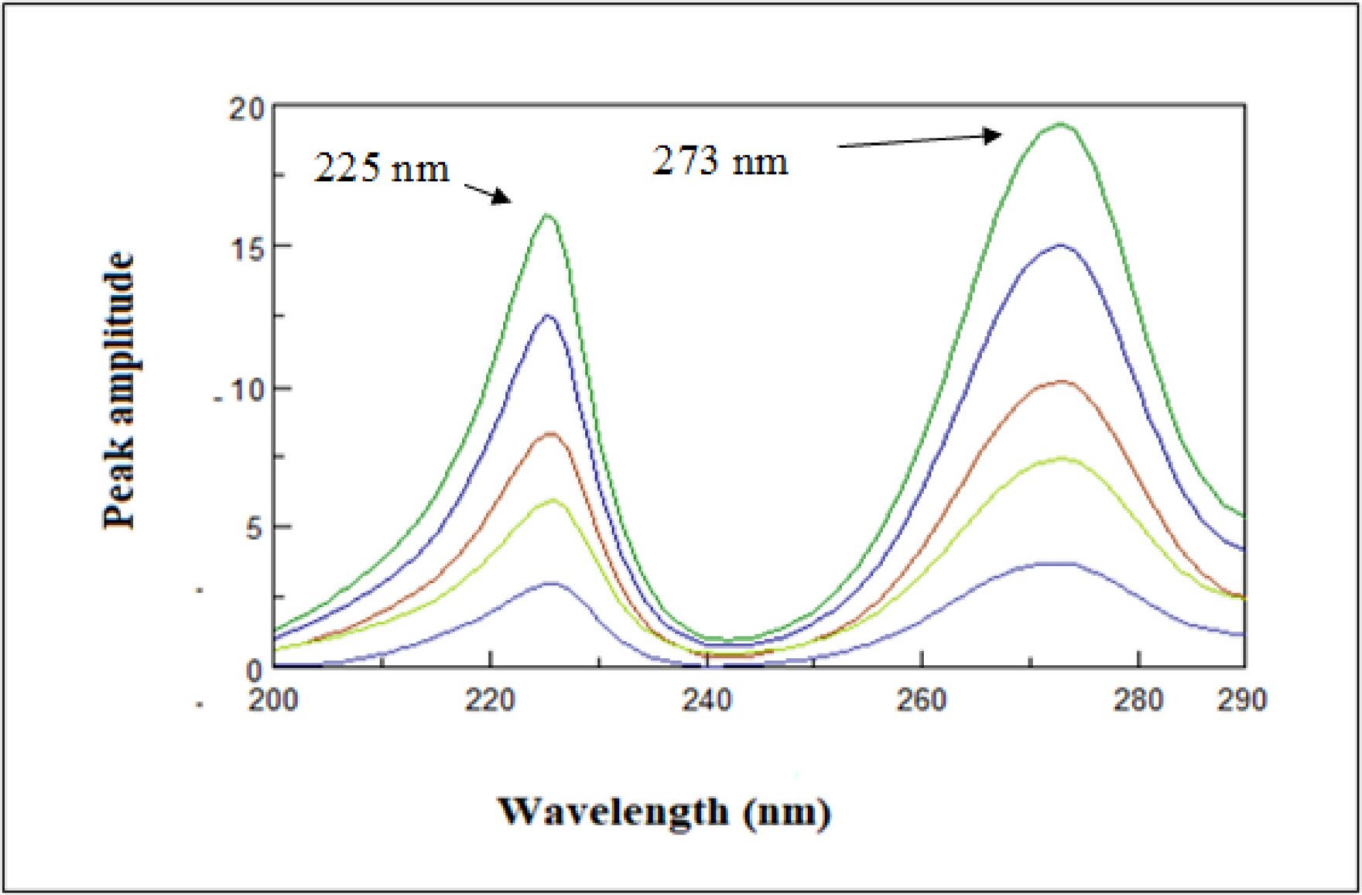
Ratio spectra of hydrochlorothiazide (3–18 µg/mL) using (5 µg/mL) of amlodipine besylate as a divisor using water as a blank.

Furthermore, the wavelength chosen should strike a compromise between great sensitivity and selective property. HYD’s ratio spectra at 273 and 225 nm were measured for peak amplitude. Difference (ΔP) to get the most accurate findings (Fig. 6).

#### First ratio derivative (RD^1^)

**Fig. 7:**
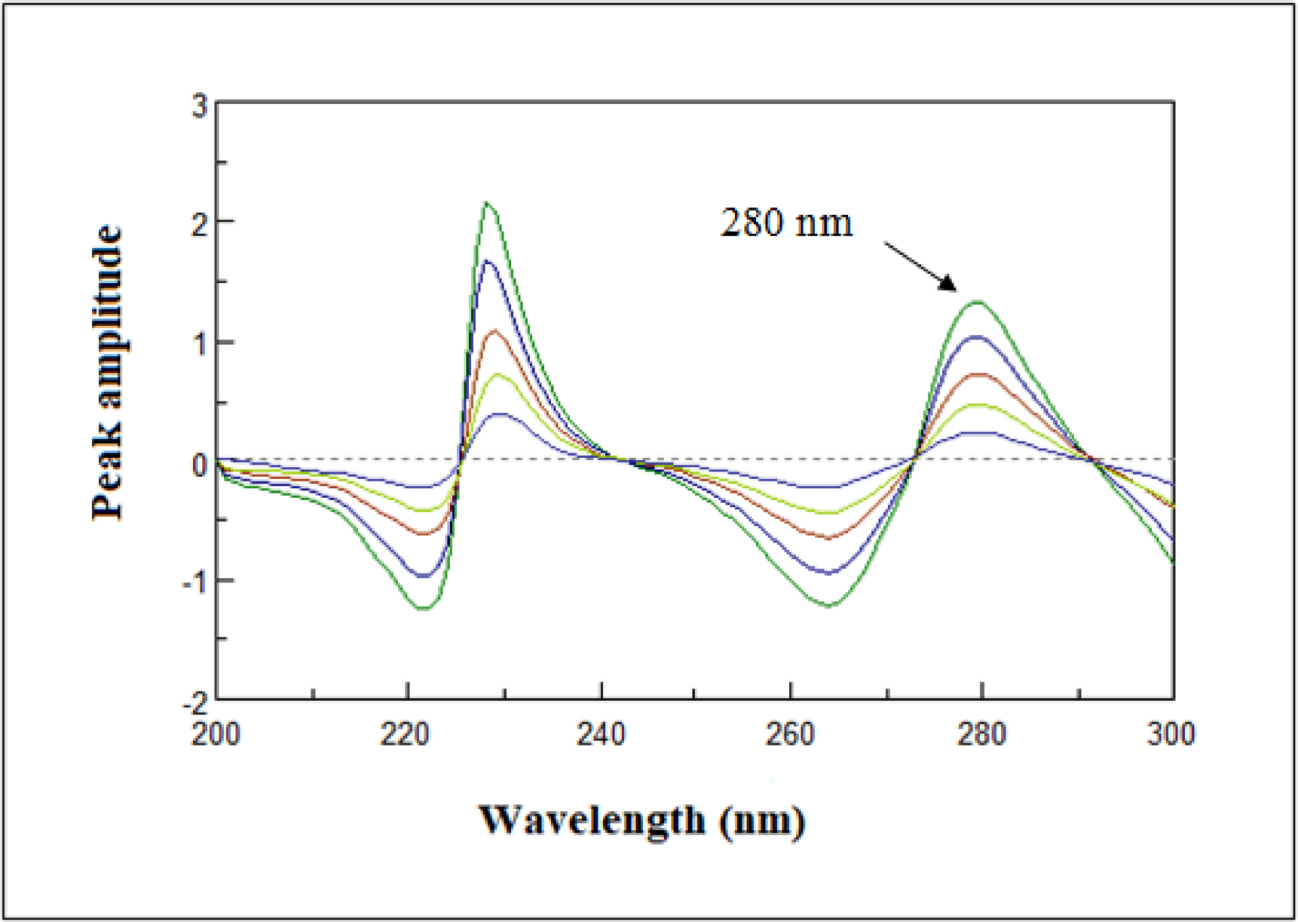
First derivative of ratio spectra of hydrochlorothiazide (3–18 µg/mL).

The mixture’s absorbed spectrum is subsequently divided by the absorbance spectra of a standard solution containing one of the its components, which acts as the divisor. The amplitudes of the generated ratio spectra show a direct correlation with the other drug’s concentration^40,41^. This technique makes it possible to use the signal’s greatest sensitivity point, whether it be a minimum or even a maximum. As shown in Fig. 7, amplitude measures were made at 280 nm in order to obtain the first derivative of the HYD spectrum.

#### Fourier self-deconvoluting (FSD)

**Fig. 8:**
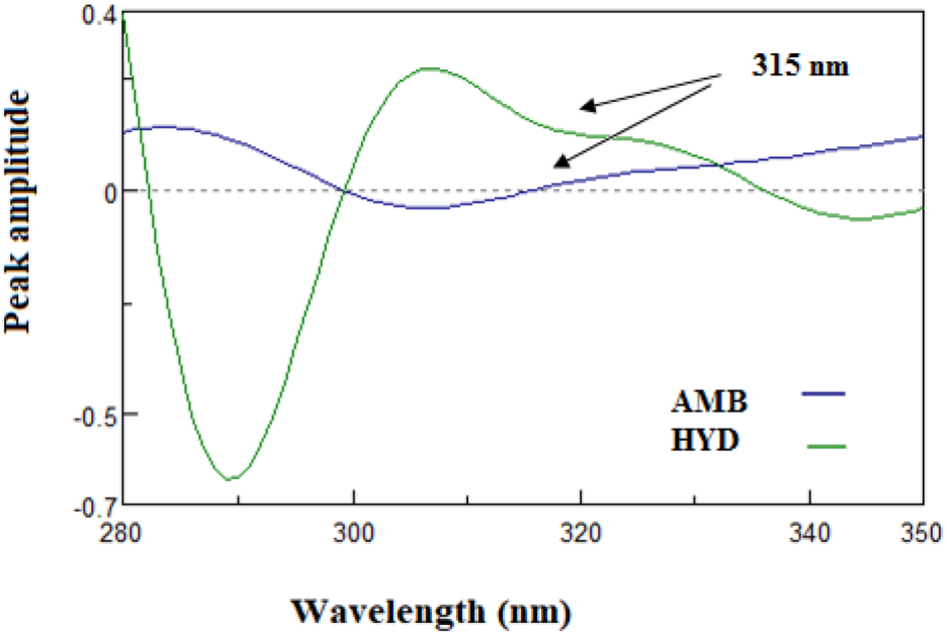
Deconvoluted spectra of HYD (10 μg/mL) determined at 315 nm zero-crossing point of AMB (10 μg/mL) deconvoluted spectrum.

The Fourier Self-Deconvolution (FSD) method is a computational technique capable of resolving severe spectral overlap by compressing their bandwidths in a manner that allows for the separation of individual components^42^. In the existence of AMB, the absorption spectrum of HYD may be ascertained by using the Fourier self-deconvolution technique. It was shown that HYD may be reliably detected at 315 nm, where AMB has no effect (zero-crossing), as Fig. 8, 9 illustrates. The Fourier deconvolution algorithm included into the spectrophotometer program was used to deconvolute the recorded zero spectra for each medication, applying a full width at half maximum (FWHM) equal to 60.

**Fig. 9:**
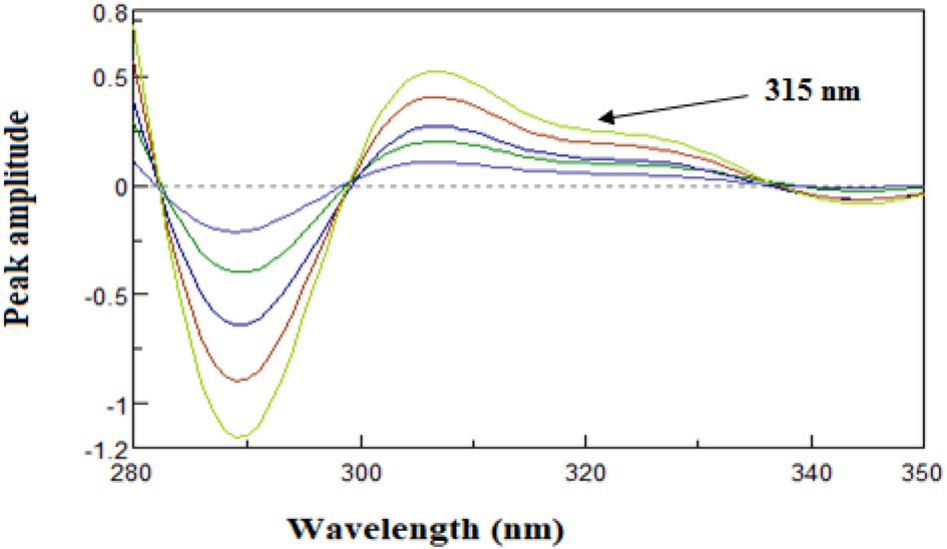
Deconvoluted spectra of hydrochlorothiazide (3–18 μg/mL) determined at 315 nm.

### Method validation

The validation of the proposed spectrophotometric methods for the simultaneous determination of AMB and HYD was systematically carried out in accordance with the guidelines established by the International Council for Harmonization of Technical Requirements for Pharmaceuticals for Human Use (ICH) ^43^. The validation process encompassed key performance characteristics, namely linearity, limit of detection, limit of quantitation, accuracy, precision (both repeatability and intermediate precision), and specificity, as detailed in Table 1.

**Table 1:** Regression parameters and validation data of determination of pure samples of AMB and HYD by the proposed spectrophotometric methods.

Parameter	AMB	HYD				
	Zero crossing	DW	D^1^	RD	RD^1^	FSD
Linearity range (µg/mL)	5–30	3–18	3–18	3–18	3–18	3–18
r	0.9992	0.9992	0.9989	0.9996	0.9993	0.9991
Slope	0.008	0.074	0.004	0.165	0.072	0.016
Intercept	0.011	0.051	0.010	0.228	0.027	0.026
LOD (µg/mL)	1.238	0.906	0.825	0.540	0.779	0.805
LOQ (µg/mL)	3.750	2.745	2.500	1.636	2.361	2.439

#### Linearity

Linearity was assessed by constructing calibration curves for each analyte over a defined concentration range. For AMB linearity was observed within the range of 5–30 µg/mL (zero-crossing method), while HYD exhibited linearity in the range of 3–18 µg/mL (for all proposed methods, dual wavelength, first derivative, ratio difference, ratio derivative and Fourier self-deconvoluting). The regression analysis demonstrated strong correlation coefficients (r values approaching unity), indicating a direct proportional relationship between absorbance or peak amplitude and concentration within the linearity range.

#### Sensitivity (the limit of detection and the limit of quantification**)**

The sensitivity of the methods was evaluated through the determination of the limit of detection (LOD) and the limit of quantification (LOQ). These parameters were calculated based on the standard deviation (SD) of the intercept of the calibration curve and the slope (S) of the regression line, using the standard ICH equations:

- LOD = 3.3 × (SD of intercept/slope).
- LOQ = 10 × (SD of intercept/slope).

These values reflect the smallest concentration of each drug that can be reliably detected or quantified using the proposed methods.

#### Accuracy

Accuracy was evaluated by analyzing three distinct concentrations of each drug, each concentration measured in triplicate. The percentage recovery (R%) was subsequently calculated by comparing the measured concentrations to the true concentrations. The obtained recovery values were found to be within the acceptable range, confirming the high accuracy of the methods As shown in Table 2.

**Table 2:** Results of accuracy and precision of determination of AMB and HYD by the proposed method.

		Parameter	Accuracy			Precision Intra-day			Precision Inter-day		
Method	Zero crossing AMB	Concentration	5	10	20	5	10	20	5	10	20
		Recovery^*^	99.167	99.360	98.611	99.167	97.870	98.621	99.167	100.230	98.620
		Mean ± SD	99.046 ± 0.389			98.553 ± 0.645			99.339 ± 0.818		
	DW HYD	Concentration	5	10	15	5	10	15	5	10	15
		Recovery^*^	99.975	98.835	101.138	99.972	99.650	100.873	99.974	100.789	101.740
		Mean ± SD	99.98 + 1.151			100.166 ± 0.634			100.835 ± 0.884		
	D^1^ HYD	Concentration	5	10	15	5	10	15	5	10	15
		Recovery^*^	100.015	101.667	99.500	99.500	101.789	99.722	100.001	98.667	98.500
		Mean ± SD	100.389 ± 1.13			100.337 ± 1.262			99.056 ± 0.822		
	RD HYD	Concentration	5	10	15	5	10	15	5	10	15
		Recovery^*^	102.667	100.932	100.791	99.500	101.789	99.722	100.000	98.667	98.500
		Mean ± SD	101.463 + 1.05			99.237 ± 1.483			100.107 ± 1.306		
	RD^1^ HYD	Concentration	5	10	15	5	10	15	5	10	15
		Recovery^*^	98.772	99.845	100.445	102.500	99.845	100.445	97.560	99.845	101.445
		Mean ± SD	99.687 + 0.848			100.93 ± 1.392			98.95 ± 2.091		
	FSD HYD	Concentration	5	10	15	5	10	15	5	10	15
		Recovery^*^	99.621	101.626	100.020	101.626	99.991	98.129	100.003	98.118	100.634
		Mean ± SD	100.416 + 10.65			99.915 ± 1.75			99.585 ± 1.309		

Recovery^*^ mean precent of three replicates.

#### Precision

The precision of the methods was examined on two levels:

- Repeatability (Intra-day precision): Three concentrations of each analyte were analyzed three times within a single day.
- Intermediate precision (Inter-day precision): The same concentrations were analyzed across three consecutive days.

The results revealed minimal variability, indicating excellent reproducibility and consistency of the measurements over time As shown in Table 2.

#### Specificity

To assess the specificity of the developed methods, synthetic binary mixtures of AMB and HYD at varying concentration ratios were prepared and analyzed. The concentration of each component in the mixtures was calculated using the respective regression equation obtained from the calibration curve. The mean percentage recoveries for both drugs in these mixtures were calculated, demonstrating that the methods can accurately and selectively quantify each drug in the presence of the other without interference. These results, presented in Table 3, confirm the high selectivity of the proposed analytical approaches.

**Table 3:** Results of laboratory prepared mixtures of AMB and HYD by the proposed method.

Method	Drug	AMB and HYD prepared mixtures (µg/mL)					Mean	SD	RSD
		10:5	5:10	10:10	20:10	10:12.5			
		Recovery%^*^							
Zero-crossing	AMB	99.167	98.526	100.478	101.025	98.134	99.466	1.245	1.251
DW	HYD	99.974	101.256	97.891	100.129	97.855	99.421	1.497	1.506
D ^1^	HYD	101.567	101.024	100.504	99.617	100.521	100.646	0.722	0.717
RD	HYD	98.857	102.014	100.781	99.821	99.443	100.183	1.239	1.237
RD ^1^	HYD	100.704	101.765	98.7641	99.254	101.471	100.391	1.331	1.326
FSD	HYD	101.52	98.641	97.951	100.874	97.975	99.392	1.686	1.696

*Average of three determinations.

### Evaluation of the environmental friendliness of proposed analytical methods

The Analytical GREEnness Calculator (AGREE), the Modified Green Analytic Procedure Index (MO-GAPI), spider diagram, and green analytical selection tool were used to assess the ecologically soundness of the steps used in the suggested methods for determining AMB and HYD. The fundamental element of the first program is the AGREE^®^ software^44^. The clock-shaped graph that AGREE offers illustrates the 12 principles of Green Chemistry, with boundaries divided into 12 parts. Each section uses a three-color system red, yellow, and green to rate how well the analytical procedure follows the GAC principle. An overall evaluation figure and color are located in the middle of the AGREE graph. The scale for the overall evaluation is from 0 to 1. Fig. S1 displays the final result (0.73) that stands for the method’s eco-friendliness; this value was arrived at by giving each of the analytical parameters a separate score.

Secondly, there was MoGAPI ^45^**. **The MoGAPI tool integrates the precise total score of the analytical Eco Scale with the advantages of GAPI’s visual impact. The software was used to ascertain if the proposed analytical processes were environmentally friendly. Offline preparation causes Sect. 1 of Fig. S2 to be red, whereas transfer, normal storage, a simple method, microextraction, and the use of green solvent cause Sects. 3–7 to be yellow. Fig. S2 shows the final MOGAPI results, which suggested that the eight pentagrams of the suggested methods had an intensified green hue. An outstanding environmentally friendly approach was shown by the total score of 84.

An assessment of the chemicals in the suggested spectrophotometric approaches was also conducted using a spider diagram to ascertain the greenness index^46^. The information used in this tool is derived from Solvent Safety Data Sheets, which provide comprehensive facts on a reagent’s properties, including its implications for safety, health, and the environment (SHE) throughout each stage of the process. In order to effectively illustrate the comprehensive sustainability of the essential compounds, a spider diagram with layers of hierarchy (Fig. S3) was developed. An analysis of five main subcategories formed the basis of this central figure. Effects on Health, General Features, Smell, Fire Safety, and Stability. The scores for each subgroup ranged from − 5 to + 5. More information is provided by secondary spider diagrams for each of the five subcategories that were emphasized in Fig. S4. Data missing from solvent safety data sheets for any of the five subcategories described earlier were treated as having no value in the calculations, as there is often not enough information available for the aforementioned compounds.

When looking for a sustainable green solvent, many industrial enterprises use the green solvent selection tool, a significant research instrument. An evaluation technique for solvents was developed by GlaxoSmithKline (GSK) using data from solvent safety data sheets^47,48^. Solvents are scored from zero to ten based on a number of separate factors, including dispersion, polarity, and hydrogen bonding (Fig. S5). In order to find the solvents that are best for the environment, chemometric equation was used whose variable is G^49^.

1 $$~G{\text{ }} = \sqrt[4]{{\left( {H{\text{ }} \times {\text{ }}S{\text{ }} \times {\text{ }}E{\text{ }} \times {\text{ }}W} \right)}}$$

In this context, G denotes the green solvent score, H signifies health, S indicates safety, E represents the environment, and W pertains to waste disposal.

In the proposed method, water underwent calculation. This is a description of the equation used to calculate the values of H, S, E, and W.:(G_water_=7.3/H_water_=9.5/S_water_=8.9/E_water_=8.9/W_water_=3.7).

The findings indicate outstanding values that signify the environmental friendliness of water as a solvent.

Moreover, the current UV spectrophotometric techniques offer distinct benefits over the previously reported chromatographic procedures and spectrophotometric methods. These include simpler execution, enhanced accuracy, and the elimination of hazardous solvents (chromatographic methods used organic solvents and reported spectrophotometric methods used sodium hydroxide as a solvent) Table S1.

### Employment of the optimized analytical techniques for the quantitative determination of AMB and HYD in pharmaceutical dosage forms

The developed analytical approaches proved to be effective for the determination of AMB and HYD in their pharmaceutical dosage forms, exhibiting excellent accuracy and no observable interference from excipients, as illustrated in Table 4.

**Table 4:** Statistical comparison between the results of determination of AMB and HYD in Amvasc plus tablets by the proposed spectrophotometric methods and results of reported methods.

Parameter	Drug							
	AMB	HYD					AMB	HYD
	Method							
	Zero-crossing	DW	D^1^	RD	RD^1^	RSD	Reported method ^29^	
N	5	5	5	5	5	5	5	5
Mean^a^	99.377	100.050	100.000	99.625	99.351	100.536	100.355	100.555
SD	0.699	0.866	1.297	2.185	1.388	1.104	0.951	1.489
Student t-test (2.306)^b^	1.853	0.656	0.628	0.786	1.323	0.02		
F	1.851	2.956	1.318	2.153	1.151	1.819		
(6.388)^b^								

^a^Mean of five determinations. ^b^The values in parenthesis are tabulated values of “*t *” and “*F *” at (*p* = 0.05). ^c^Reported method: by simultaneous equation method based on measurement of absorbance at 238 nm and 271 nm.

### Standard addition technique

A recovery study was conducted using the standard addition method, in which varying concentrations of pure AMB and HYD were added to previously analyzed pharmaceutical formulations. The mean percentage recovery ± relative standard deviation (RSD%) of the spiked concentrations was calculated, as presented in Table 5, indicating the absence of matrix interference.

**Table 5:** Results of analysis of AMB and HYD in Amvasc plus tablets by the proposed methods and by applying the standard addition technique.

Drug	Pharmaceutical	Pure added (µg/mL)		Methods					
				Zero-crossing	DW	D^1^	RD	RD^1^	FRD
				Pure found %					
AMB	10 mg	5		100.46					
		10		101.97					
		15		101.447					
Mean				101.292					
SD				0.767					
HYD	12.5 mg	3			100.327	99.913	99.667	101.367	100.467
		6			101.757	100.400	99.233	98.317	100.800
		9			98.478	100.500	101.156	100.044	99.867
Mean					100.187	100.271	100.019	99.909	100.378
SD					1.644	0.314	1.008	1.529	0.473

### Statistical evaluation

A comprehensive statistical analysis was conducted to evaluate and compare the performance of the developed analytical methods against each other and in relation to reported method^29^. The assessment involved multiple statistical tools to ensure the robustness and reliability of the comparison.

Initially, both the Student’s *t*-test and the F-test were employed to examine potential differences in accuracy and precision between the proposed methods and the reference technique^29^. The obtained *t* and *F* values were found to be lower than their corresponding critical values, signifying the absence of statistically significant differences between the methods, as presented in Table 4.

In addition, a one-way Analysis of Variance (ANOVA) was applied at a 95% confidence level to further validate the consistency of the results across the different analytical approaches. The ANOVA outcomes (Table 6) confirmed that there were no meaningful variations among the evaluated methods.

**Table 6:** ANOVA analysis for determination of HYD and AMB in their dosage form by proposed methods and reported method.

	Source of Variation	Sum of squares	Degree of freedom	Mean squares	F-value	*P*-value	F critical
HYD	Between Groups	4.860	5.000	0.972	0.464	0.799	2.621
	Within Groups	50.274	24.000	2.095			
	Total	55.134	29.000				
AMB	Between Groups	1.000	2.388	3.429	0.101	5.318	2.388
	Within Groups	8.000	0.696				5.572
	Total	7.960	9.000				

To reinforce the findings from the ANOVA test, Tukey’s Honest Significant Difference (HSD) test was utilized as a post-hoc analysis tool. This method enabled the detection of any potential differences in group means with enhanced sensitivity. The visual representation in Fig. S6 illustrates the confidence intervals for each data group, with horizontal lines and central markers indicating the group means. The observed overlap between intervals further supports the conclusion that no statistically significant differences exist among the tested methods.

## Conclusion

One spectrophotometric method for determining AMB and five for determining HYD are available; they are sensitive, accurate, and inexpensive; and they are ideal for usage in labs that do not have access to the resources needed for chromatographic procedures. Moreover, these approaches have the benefit of being ecologically sustainable, unlike the described method that employs environmentally detrimental solvents like sodium hydroxide. The suggested methodologies were effectively used for the quantification of AMB and HYD in bulk powder, laboratory-prepared mixes, and pharmaceutical formulations. The ratio difference method is highly effective for determining HYD because of its low limit of detection (LOD) and limit of quantification (LOQ). Statistical analysis was done using Student’s t-test, F-test, and ANOVA, and neither the reported method nor the proposed methods differed significantly from each other. In addition, the ANOVA findings were double-checked using Tukey’s HYD test.

In the end, the suggested approaches were found to be very eco-friendly after an evaluation utilizing the AGREE, MOGAPI, spider chart, and green analytical selection tools.

## Supplementary Information

Below is the link to the electronic supplementary material.

Supplementary Material 1

## Data Availability

All data generated or analyzed during this study are included in this published article.
