# Supplementary material for: Spectroscopic methods for the simultaneous determination of amlodipine besylate and Hydrochlorothiazide in their binary mixture and pharmaceutical dosage form
Source: Sci Rep. 2026 Feb 2;16:4541. doi: 10.1038/s41598-025-33191-4 (PMC12868856; doi:10.1038/s41598-025-33191-4)
Supplement: Supplementary file 1 — Supplementary Material 1 [file 41598_2025_33191_MOESM1_ESM.docx]

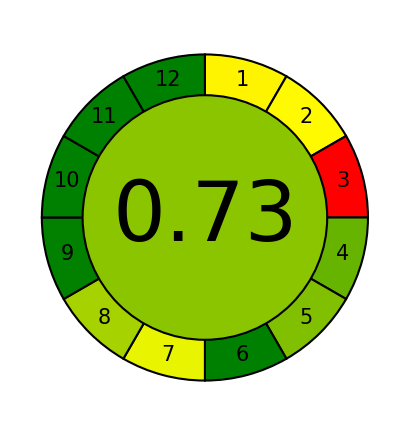


**Figure.S1.** Analytical greenness score calculation for proposed spectrophotometric methods.


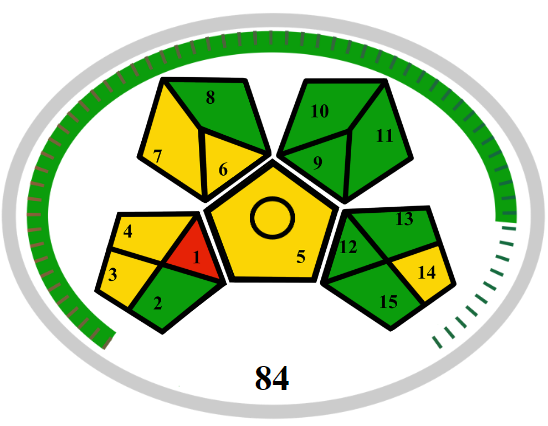


**Figure .S2**. The suggested spectrophotometric procedures evaluation using the Modified Green Analytical procedure index (MOGAPI).


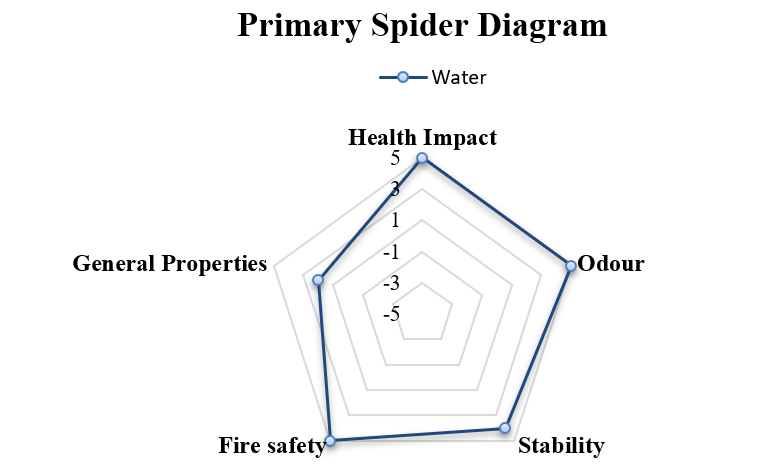


**Figure.S3**. A primary spider chart exhibiting water properties.


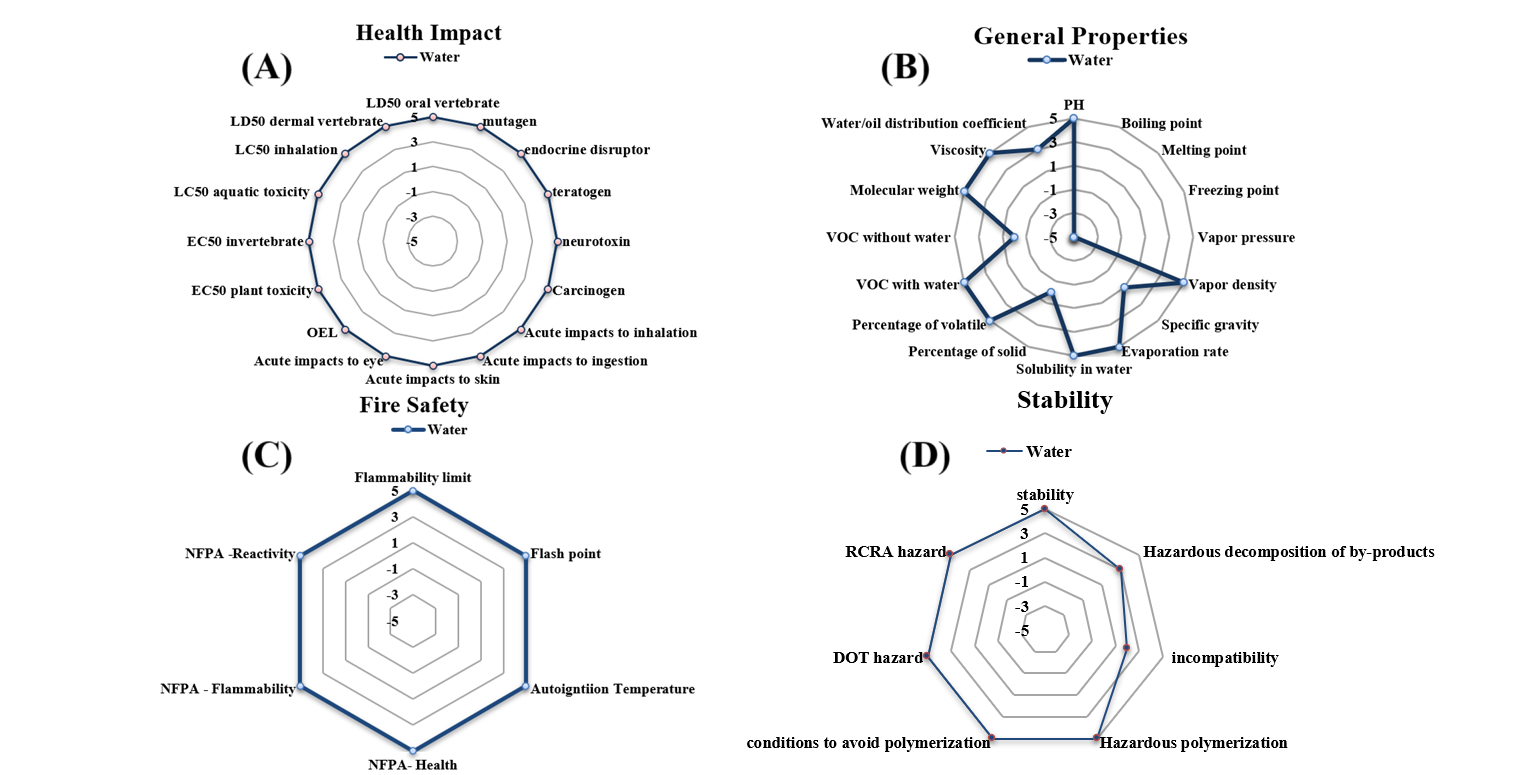


**Figure.S4.** Supplemental water charts for (A) potential health risks, (B) general features, (C) fire protection, and (D) stability.


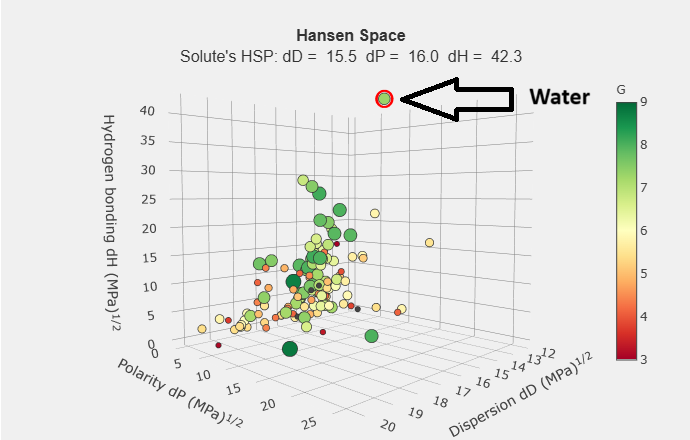


**Figure.S5.** Green analytical selection tool evaluation for water


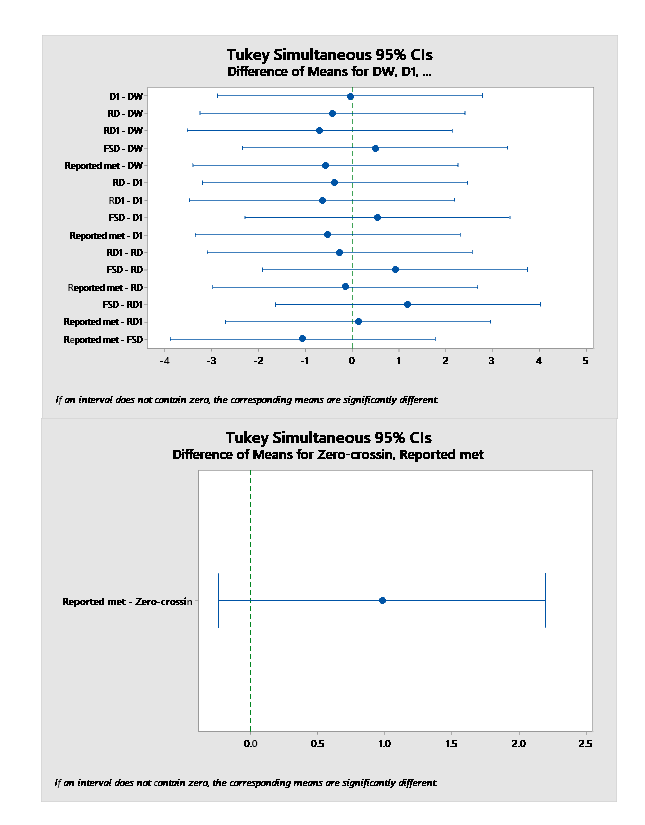


A

B

**Figure. S6.** Tukey’s significant difference plot for (A) hydrochlorothiazide (B) amlodipine besylate.

Table 1S: Comparison between proposed and published methods.

| **Published chromatographic method [18]** | **Published spectrophotometric method [31]** | **Published spectrophotometric method [30]** | **Published spectrophotometric method [29]** | **Published spectrophotometric method [20]** | **Proposed methods** | **Methods** |
| --- | --- | --- | --- | --- | --- | --- |
| Acetonitrile: water (0.1 % trifluoroacetic acid) (70:30 v/v) | Methanol: water (50:50v/v) | Methanol | 0.1 N sodium hydroxide | Methanol | Water | Solvent |
| AMB: 30-250 µg/mL  HYD: 35-285 µg/mL | AMB:2.5-17.5  HYD: 2.5-17.5 | AMB: 2.5-50 µg/mL  HYD: 1-20 µg/mL | AMB: 5-30 µg/mL  HYD: 2.5-15 µg/mL | AMB: 0.25-1.25 µg/mL  HYD: 6-30 µg/mL | AMB: 5-30 µg/mL  HYD: 3-18 µg/mL | Linearity |
| AMB: 7.5 µg/mL  HYD: 9.5 µg/mL | Not reported | Not reported | Not reported | Not reported | AMB: 1.238 µg/mL  HYD: DW: 0.906, D^1^: 0.825, RD: 0.540, RD^1^: 0.779, FSD: 0.805 µg/mL | LOD |
| AMB: 22.5 µg/mL  HYD: 28.5 µg/mL | Not reported | Not reported | Not reported | Not reported | AMB: 3.750 µg/mL  HYD: DW: 2.745, D^1^: 2.500, RD: 1.636, RD^1^: 2.361, FSD: 2.439 µg/mL | LOQ |
| 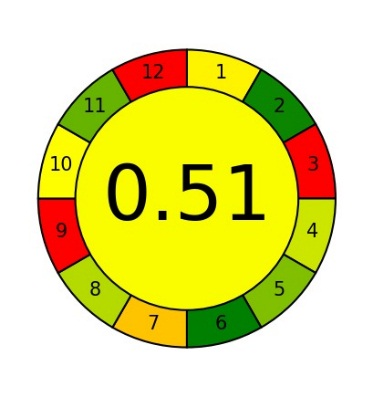 | 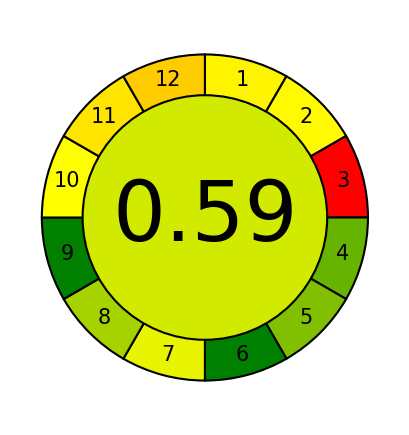 | 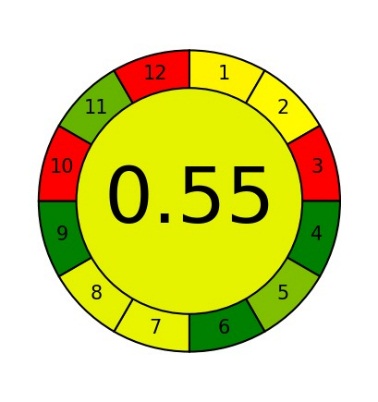 | 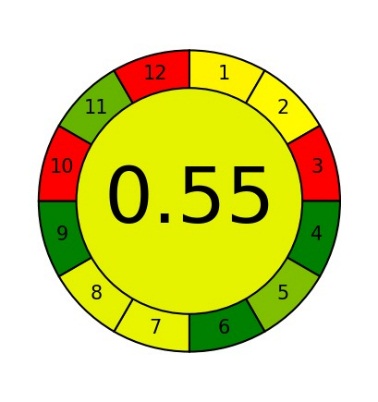 | 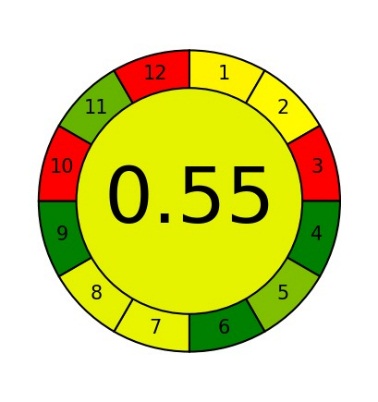 | 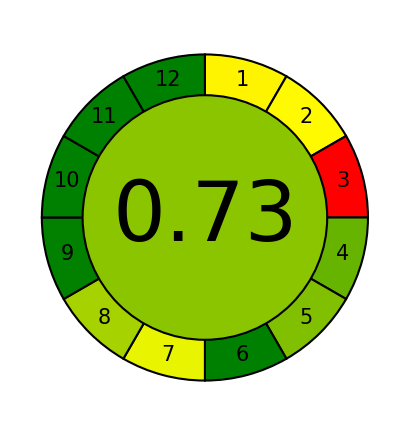 | AGREE |
